# Multi-target mechanisms and potential applications of quercetin in the treatment of acne vulgaris

**DOI:** 10.3389/fphar.2025.1523905

**Published:** 2025-04-07

**Authors:** Yang Bo, Yiming Li

**Affiliations:** ^1^ Department of Dermatology, Sichuan Second Hospital of T.C.M, Chengdu, Sichuan, China; ^2^ Department of Dermatology, Institute of Traditional Chinese Medicine of Sichuan Academy of Chinese Medicine Sciences, Chengdu, Sichuan, China

**Keywords:** quercetin, acne, mechanisms, pharmacology, inflammation

## Abstract

Acne vulgaris, a prevalent inflammatory dermatosis, afflicts approximately 90% of adolescents globally. Despite the efficacy of conventional therapies, including antibiotics and retinoids, their use is frequently limited by adverse effects and the emergence of drug resistance. Quercetin, a naturally occurring flavonoid, has garnered significant attention owing to its diverse biological activities, encompassing anti-inflammatory, antioxidant, antimicrobial, and immunomodulatory properties. This review comprehensively explores the multi-target mechanisms of quercetin in the treatment of acne, focusing on its ability to modulate inflammatory cytokine production, oxidative stress pathways, sebaceous gland activity, and microbial populations. Additionally, quercetin promotes skin barrier repair and reduces post-inflammatory hyperpigmentation and scarring through its antioxidant and anti-fibrotic effects. Despite promising *in vitro* and preclinical findings, challenges such as quercetin’s low bioavailability and lack of robust clinical evidence necessitate further research. Advanced delivery systems, including nanoparticles and combination therapies, may optimize its therapeutic potential. This review provides insights into the molecular mechanisms and clinical applications of quercetin, highlighting its potential as a safe and effective alternative for acne management.

## 1 Introduction

Acne vulgaris is a chronic inflammatory disorder of the follicular sebaceous gland unit, with a multifactorial etiology. Genetic predisposition, androgen-driven hypersecretion of sebum, abnormal keratinization of follicular ducts, proliferation of *Cutibacterium acnes* (*C. acnes*), and immune-inflammatory responses are all implicated in its pathogenesis (Layton and Ravenscroft, 2023; Hazarika, 2021; Melnik, 2023; Zouboulis et al., 2014; Byrd et al., 2018). The disease typically manifests in sebaceous gland-rich areas such as the cheeks, forehead, and back, presenting with polymorphic lesions including comedones, papules, pustules, nodules, and cysts. These can lead to persistent scarring and hyperpigmentation, significantly impacting aesthetic appearance and quality of life, with a high propensity for recurrence (Harper, 2020). Currently, the global standard of care for acne involves topical retinoids, benzoyl peroxide, antibiotic ointments, and systemic therapies such as oral antibiotics and anti-androgens (Oge et al., 2019; Leyden et al., 2017; Chlebus et al., 2019; Eichenfield et al., 2021). However, these treatments are often associated with adverse effects, including skin irritation, numerous contraindications, variable efficacy, the potential for resistance, and teratogenic risks, thereby limiting their clinical utility (Habeshian and Cohen, 2020). In recent years, natural products derived from plants have garnered increasing attention for their efficacy and lower toxicity. Extensive research indicates that botanical drugs, rich in pharmacologically active metabolites, hold significant promise for the treatment of acne (Chen et al., 2016).

Quercetin is a natural flavonoid metabolite widely present in plants in the form of glycosides, characterized by its yellow needle-like crystals with a stable chemical structure (Cui et al., 2017), which enables its participation in a diverse array of biological processes. Renowned for its potent antibacterial (Qi et al., 2022), antiviral (Di Petrillo et al., 2022), anti-inflammatory (Guan et al., 2021), and antioxidant properties (Jeon et al., 2019), quercetin has garnered increasing attention for its therapeutic potential. Recent research has highlighted its efficacy in the treatment of various diseases, including cardiovascular disorders (Zhou et al., 2022), cancer (Slika et al., 2022), diabetes (Dhanya, 2022), and dermatological conditions (Zaborowski et al., 2024). The pathogenesis of acne is now widely understood to involve four key factors: excessive sebum production, abnormal keratinization of follicular sebaceous ducts, colonization by *C. acnes*, and subsequent inflammation. Quercetin, through its multifaceted bioactive properties, has shown promise in addressing these underlying mechanisms. Therefore, we have reviewed the possible molecular mechanisms and potential applications of quercetin in the treatment of acne, providing a reference for future research and clinical applications.

## 2 Methods of data acquisition

To comprehensively assess the potential molecular mechanisms by which quercetin may treat acne, we conducted a systematic literature search for studies published since January 2003 in PubMed, Web of Science, Embase, and the China National Knowledge Infrastructure. The search terms included “quercetin,” “bioavailability of quercetin,” “pharmacokinetics of quercetin,” “acne,” “inflammation,” “oxidative stress,” “*C. acnes*,” “microbiota,” “sebaceous glands,” “immunity,” “skin barrier,” “melanin,” and “scarring.” Boolean operators (AND/OR) were employed to efficiently combine these terms, ensuring a comprehensive and accurate search. Additionally, reference lists and relevant records were reviewed to identify additional studies. Inclusion criteria were as follows: 1) studies investigating quercetin’s role in acne treatment, including its bioavailability, pharmacokinetics, and therapeutic potential; 2) studies exploring quercetin’s effects on acne-related pathological mechanisms, such as inflammation, oxidative stress, immune modulation, sebaceous gland function, and skin barrier integrity; 3) studies evaluating quercetin’s impact on microbiota, melanin metabolism, and scar formation. Studies unrelated to quercetin or acne were excluded. During the screening process, we initially reviewed titles and abstracts to identify relevant studies that met the inclusion criteria. Full-text articles were then retrieved for a detailed evaluation, focusing on the extraction of data related to quercetin’s therapeutic mechanisms, its pharmacological actions in acne, and its interactions with inflammation, oxidative stress, sebaceous glands, microbiota, skin barrier, melanin, and scarring. Finally, the synthesized data were analyzed to provide a comprehensive understanding of the molecular mechanisms through which quercetin may treat acne, while discussing the limitations of existing research and potential future research directions. This review aims to provide a theoretical foundation for further studies on quercetin as a treatment for acne.

## 3 Biological characteristics of quercetin

### 3.1 Source and physicochemical properties of quercetin

Quercetin is a flavonol subclass of flavonoids, with the chemical name 3,3′,4′,5,7-pentahydroxy flavone and a molecular formula of C_15_H_10_O_7_, corresponding to a relative molecular mass of 302.236 g/mol (Yang et al., 2020). It appears as a yellow powder or needle-like crystals, insoluble in water but soluble in alkaline aqueous solutions (Li et al., 2016a). When dissolved in ethanol, quercetin exhibits a distinctly bitter taste, and upon thermal decomposition, it releases a pungent odor. In the diet, quercetin is typically found in its glycoside form, with particularly high concentrations in vegetables such as onions and tomatoes, as well as in fruits like apples, cherries, and various berries (Wan et al., 2021). Additionally, quercetin is abundant in several botanical drugs, including *Ginkgo biloba L*., *Hypericum perforatum L.*, and *Sambucus nigra L.* (Li et al., 2016b).

### 3.2 Pharmacokinetics of quercetin

Upon oral administration, quercetin initially interacts with salivary proteins, forming soluble aggregates through hydrogen bonding and hydrophobic interactions. After reaching the stomach, a portion of quercetin is degraded into phenolic acids, which are subsequently absorbed by the gastric epithelium. In the small intestine, quercetin undergoes phase II metabolism, catalyzed by uridine diphosphoglucuronyl transferase (UGT) for glucuronidation, sulfotransferase (SULT) for sulfation, and catechol-O-methyl transferase (COMT) for methylation, generating various metabolites such as quercetin-3-glucuronide, quercetin-3′-sulfate, and isorhamnetin-3-glucuronide. These metabolites are then absorbed by enterocytes via passive diffusion (Zhu et al., 2024). Following absorption, quercetin and its metabolites are transported to the liver for further biotransformation. After hepatic metabolism, the metabolites are exported to the circulation and bile through multidrug resistance-associated proteins (MRP), with biliary metabolites either recirculating to the small intestine or being excreted in the feces (Luca et al., 2020). In the colon, unmetabolized quercetin from the small intestine and liver undergoes microbial-mediated biotransformation, including demethylation, hydroxylation, deglycosylation, decarboxylation, and ring cleavage, resulting in the formation of low-molecular-weight phenolic metabolites that are more readily absorbed (Wang et al., 2023). Ultimately, quercetin and its metabolites are excreted through expired carbon dioxide, feces, and urine, primarily in the form of glucuronide or sulfate conjugates.

### 3.3 Bioavailability of quercetin

Early studies reported that the oral bioavailability of quercetin is notably low (around 2%). Subsequent research using radiolabeled quercetin aglycone measured the absolute bioavailability at approximately 44.8% (Walle et al., 2001). Human studies have shown that quercetin has a high oral clearance rate (3.5 × 10^4^ L/h), with a half-life ranging from 11 to 28 h, and an average terminal half-life of 3.5 h (Konrad and Nieman, 2015). The primary plasma metabolites are quercetin-3′-sulfate and quercetin-3-glucuronide, which reach peak levels at 0.8 h and 0.6 h, respectively (Dabeek and Marra, 2019). Quercetin’s bioavailability is influenced by factors such as low absorption efficiency, hepatic first-pass metabolism, and gut microbiota-mediated metabolism, with notable individual variability. These variations are attributed to both endogenous factors (e.g., sex, age, and intestinal permeability) and exogenous factors (e.g., food matrix, dietary fat, and quercetin glycosylation). To enhance its bioavailability, researchers are exploring various drug delivery systems and dietary adjuncts, such as nanoparticles and liposomes, to improve quercetin absorption and systemic stability (Tomou et al., 2023).

### 3.4 Toxicological studies and safety of quercetin

In 2010, the Food and Drug Administration in the United States confirmed quercetin (GRN 000341) as a Generally Recognized as Safe (GRAS) ingredient for use in beverages, cereal products, pasta, processed fruits and juices, and gummy beverages up to a maximum of 500 mg per serving (US Food and Drug Administration, 2010). The European Union recognized quercetin as a new food ingredient in its 2015 response document (European Parliament and Council of the European Union, 2015). Quercetin is also available as a food additive in Japan and Korea. Clinical studies have shown that the use of doses of up to 1,000 mg/day for several months did not adversely affect hematological parameters, liver and kidney function, or serum electrolyte levels, indicating that it is well tolerated in humans (Andres et al., 2018). Animal and human genotoxicology studies have consistently confirmed that oral quercetin has no significant acute toxicity or mutagenic/carcinogenic risk (Okamoto, 2005; Rauf et al., 2018; Stoner, 1991). Its almost complete metabolism in the gut and liver in first-pass metabolism further reduces systemic toxicity (Khursheed et al., 2020; Orrego-Lagarón et al., 2016). However, the potential risks still need to be evaluated with caution. Studies have shown that the toxicity of quercetin may be related to reactive oxygen metabolites produced during the antioxidant process, suggesting that the safety of long-term use needs to be evaluated in chronic diseases (Harwood et al., 2007). Quercetin may aggravate the toxicity of digoxin in patients with cardiac insufficiency, induce hypotension or coagulation disorders, and affect fetal growth (Mieszkowski et al., 2011; Pérez-Pastén et al., 2010). In conclusion, although the benefit-risk ratio for quercetin remains favorable for most populations, targeted preventive measures, including dose moderation and clinical monitoring, are necessary for susceptible subgroups and for situations involving high-dose, prolonged administration.

Building upon the understanding of quercetin’s physicochemical properties, pharmacokinetics, bioavailability toxicological studies and safety, it is crucial to explore its potential therapeutic applications. Given its widespread presence in the diet and its diverse metabolic pathways, quercetin has garnered attention for its multifaceted biological activities. These properties position quercetin as a promising candidate for the treatment of various diseases, including inflammatory conditions such as acne vulgaris. In the following section, we delve into the multi-target mechanisms through which quercetin exerts its therapeutic effects in acne vulgaris, shedding light on its potential as an effective adjunct or alternative treatment.

## 4 Multi-target mechanisms of quercetin in acne vulgaris treatment

### 4.1 Inflammation regulation effects of quercetin

#### 4.1.1 Inhibition of inflammatory cytokines

Acne vulgaris, an inflammatory skin disorder, is closely linked to the production of multiple pro-inflammatory cytokines by mononuclear macrophages. Key mediators such as interleukin-6 (IL-6), IL-1α, IL-8, and tumor necrosis factor-α (TNF-α) play pivotal roles in triggering the innate immune response, thereby promoting follicular hyperkeratosis and the characteristic inflammatory lesions of acne. One study demonstrated the presence of IL-1 and TNF-α in papule biopsies from patients with inflammatory acne vulgaris (Thanh et al., 2022). The heightened immunoreactivity of IL-1β in these tissues, along with its positive correlation with disease severity, suggests that IL-1β could serve as a promising target for novel therapeutic interventions. Another experimental study confirmed that quercetin exerts inhibitory effects on cytokines TNF-α, IL-1β, and IL-6, demonstrating its capacity to suppress LPS-induced inflammation and oxidative stress in macrophages (Tang et al., 2019). This effect is attributed to quercetin’s chemical stability and its potent antioxidant and anti-inflammatory properties. This is consistent with the findings of Thanh et al. (2022) and provides a basis for developing new therapeutic strategies. However, the study did not explore the effects of long-term use of quercetin, including potential dose-dependent side effects and optimal dose determination. Furthermore, quercetin also promotes the production of anti-inflammatory factors such as transforming growth factor-β (TGF-β), which helps modulate the inflammatory microenvironment and supports the repair of acne lesions (Lim et al., 2021; Ghafouri-Fard et al., 2021b). This conclusion, however, requires further experimental validation, particularly regarding the specific mechanisms by which quercetin modulates the TGF-β signaling pathway.

#### 4.1.2 Regulation of inflammatory signaling pathways

Current hotspots of research on acne inflammatory signaling pathways include MAPK/NF-κB (Cha et al., 2024), PI3K/AKT (Roy et al., 2023), TGF-β/Smad (Moon et al., 2019), and Wnt/β-catenin signaling pathways (Huang et al., 2019). Quercetin can intervene in multiple inflammatory signaling pathways. It has been found that quercetin inhibited the expression of inflammatory mediators in human immortalized keratinocytes (HaCaT) cells and human dermal microvascular endothelial cells (HDMECs) by directly interacting with p65 and intercellular cell adhesion molecule-1 (ICAM-1), thereby blocking NF-κB signaling and ICAM-1 expression (Meng et al., 2024). This finding provides evidence at the molecular mechanism level for quercetin as a potential anti-inflammatory therapeutic agent. While, the specific binding sites between quercetin and its targets require further investigation. Several studies have shown that quercetin effectively suppresses the production of IL-1β, IL-6, IL-8, and TNF-α in *C. acnes*-stimulated HaCaT, human myeloid leukemia mononuclear cells (THP-1), and mouse leukemia cells of monocyte macrophage (RAW 264.7) by inhibiting key inflammatory signaling pathways, including PI3K/Akt, MAPK, and NF-κB. Quercetin also reduces the phosphorylation of MAPK subfamilies ERK, JNK, and p38 in *C. acnes*-stimulated HaCaT and THP-1, thereby significantly attenuating the inflammatory response in *C. acnes*-induced acne models (Lim et al., 2021; Ghafouri-Fard et al., 2021b). Additionally, quercetin inhibits the transcriptional activity of NF-κB and activator protein-1 (AP-1), further mitigating inflammation.

The aforementioned studies demonstrate that quercetin exhibits broad anti-inflammatory potential by suppressing the expression of various inflammatory mediators and modulating inflammatory signaling pathways. Its mechanism of action is closely linked to the pathogenesis of acne inflammation, and this has been validated in multiple studies, indicating a high level of potential efficacy. Nevertheless, current research still presents certain limitations. For instance, due to the complexity of quercetin’s mechanisms of action, such as non-specific effects arising from its polyphenolic structure that may confound experimental results, findings from *in vitro* or high-concentration models require careful interpretation. Additionally, there is insufficient systematic clinical trial data to substantiate its specific action pathways and therapeutic outcomes. Consequently, future investigations should prioritize more rigorous *in vivo*, *in vitro*, and clinical studies to further elucidate its targeted anti-inflammatory mechanisms and refine its application strategies in acne treatment.

#### 4.1.3 Regulation of Th cell function

The immune response in acne is intricately linked to alterations in the *C. acnes* phylotype, gene pool, and transcriptional activity (Jin et al., 2023). Adaptive immune cells, particularly Th1 and Th17 lymphocytes, play a critical role in acne pathogenesis. Recent *in vitro* studies have demonstrated that *C. acnes* can stimulate adaptive immune responses by inducing the secretion of pro-inflammatory cytokines (Moreno-Arrones and Boixeda, 2016), such as IFN-γ and IL-17A, from Th1 and Th17 lymphocytes through Toll-like receptors (TLRs) (Ozlu et al., 2016), activation of inflammasomes (Moreno-Arrones and Boixeda, 2016), induction of matrix metalloproteinases (MMPs) (Hammam et al., 2020), and stimulation of antimicrobial peptide (AMP) activity (Harder et al., 2013). *C. acnes* activates monocytes via the TLR-2 signaling pathway, leading to increased IL-12 production, which in turn activates Th1 and Th17 cells, promoting the secretion of IFN-γ and IL-17 and enhancing the inflammatory response (Huang et al., 2024). Additionally, IL-17+ cells have been identified in perifollicular infiltrates of inflammatory acne biopsy specimens, underscoring the role of Th17 cells in acne (Agak et al., 2018). Cytokines such as IL-1β, IL-6, and TGF-β, which are crucial for Th17 activation, have also been detected in acne lesions, reinforcing the classification of acne as a Th17-mediated disease (Kistowska et al., 2015). Quercetin has been shown to exert inhibitory effects on the key innate immune receptor TLR-2, thereby modulating the innate immune system.25 It has also been shown that quercetin has a modulatory effect on T-cell activation, with its ability to enhance IFN-γ secreted by Th1 cells and inhibit the Th-2-derived cytokine IL-4, which contributes to the enhancement of Th1 cell-mediated immune response (Li et al., 2016b). IFN-γ, produced by Th1 cells, has the capacity to inhibit Th17 differentiation and function. By enhancing Th1 cell responses, quercetin may relatively suppress Th17 hyperactivity. Consequently, quercetin can modulate the Th1/Th2 balance, inhibit Th17 cell activation, and reduce inflammatory responses. Through its effects on immune cell function, quercetin improves the skin’s immune environment, thereby alleviating the inflammatory manifestations of acne. While these studies provide valuable mechanistic insights, most remain in the early stages and fail to fully replicate the complexities of clinical environments. In particular, there is a lack of sufficient clinical data regarding the specific roles of immune cell subsets and how these may differ across individuals. The relationship between Th1 and Th17 responses may exhibit complex duality in various diseases and individuals. Whether quercetin’s modulation of this immune balance could negatively affect other immune functions, particularly in terms of disrupting immune tolerance with long-term use, requires further investigation.

### 4.2 Antioxidant effects

Acne lesions are characterized by a significant local state of oxidative stress, marked by an imbalance between the excessive generation of reactive oxygen species (ROS) and the skin’s antioxidant defense mechanisms. This oxidative stress not only exacerbates the inflammatory response within follicular sebaceous gland units but also promotes apoptosis and the release of MMPs, further compromising skin barrier integrity (Bungau et al., 2023). Quercetin, a potent antioxidant, directly scavenges ROS, mitigating oxidative damage to skin cells (M et al., 2023). Additionally, quercetin enhances cellular antioxidant defenses by upregulating the expression of key antioxidant enzymes, such as superoxide dismutase (SOD) and glutathione peroxidase (GSH-Px), thereby protecting skin cells from oxidative injury (Xu et al., 2019; Zhou et al., 2023; Alizadeh and Ebrahimzadeh, 2022). Studies have confirmed that quercetin and its derivative dihydroquercetin effectively prevent ROS formation, glutathione (GSH) depletion, caspase-3 activation, and increase the levels of antioxidant proteins such as heme oxygenase-1 (HO-1), NAD(P)H:quinone oxidoreductase 1 (NQO1), and catalase (CAT). These actions significantly reduce UVA-induced damage to skin fibroblasts and epidermal keratinocytes, thereby alleviating oxidative stress in acne lesions, facilitating lesion regression, and promoting skin repair (Rajnochová Svobodová et al., 2022). Tang et al. (2019) investigated quercetin’s impact on inflammatory responses and oxidative stress in lipopolysaccharide-induced RAW264.7 cells through *in vitro* evaluation and theoretical modeling. Their findings suggest that the oxygen atom on quercetin’s B ring is the primary site of electron density change, underpinning its ROS-scavenging activity. Furthermore, Another study demonstrated that quercetin 3-O-β-D-glucuronide exerts protective effects on the skin, including anti-inflammatory and antioxidant actions against UVB- and H_2_O_2_-induced oxidative stress (Ha et al., 2021). Quercetin also reduces the expression of pro-inflammatory genes (COX-2, TNF-α) in stressed HaCaT cells, increases NRF2 expression, and inhibits melanin production in α-MSH-treated mouse melanoma (B16F10) cells, indicating its potential as a natural whitening and antioxidant agent in skincare formulations (Lu et al., 2019). In summary, quercetin’s antioxidant effects in acne primarily involve scavenging free radicals, inducing antioxidant enzyme synthesis, and mitigating UV-induced damage. These mechanisms collectively contribute to reducing the inflammatory response and skin damage associated with acne. Although quercetin has shown promising antioxidant effects in *in vitro* experiments, it is prone to non-specific interactions in such settings. Its specific mechanism of action in the clinical setting is still not fully explored. Key questions such as how quercetin acts in skin cells, whether its activity is dose-dependent, and how it interacts with other cytokines require further clinically controlled studies to validate its effectiveness in treating acne.

### 4.3 Sebum-regulating effects of quercetin

#### 4.3.1 Inhibition of sebaceous gland cell proliferation and lipid synthesis

Excessive sebaceous gland activity is a primary contributor to the development of acne. Research indicates that the sebum of acne patients undergoes qualitative changes, characterized by increased squalene production and reduced levels of free fatty acids (Pappas et al., 2009; Plewig and Kligman, 2000). This alteration in sebum composition dilutes the concentration of linoleic acid, leading to a relative deficiency that contributes to acne formation. Additionally, pro-inflammatory sebum lipid fractions, including monounsaturated fatty acids (MUFA) and lipoperoxides, play a significant role in acne pathogenesis (Clayton et al., 2019). Quercetin has been shown to regulate lipid metabolism via the PI3K/Akt signaling pathway, thereby mitigating sebum overproduction—a critical factor in acne control (Fitz-Gibbon et al., 2013). Furthermore, quercetin influences lipid metabolism pathways within sebaceous gland cells, modulating the expression of lipid metabolism-related factors such as sterol regulating element binding protein 1c (SREBP-1c) and peroxisome proliferator-activated receptor-γ (PPAR-γ). This regulation improves the composition and properties of sebum, facilitating its discharge from the hair follicle and preventing clogging (Saleh Al-Maamari et al., 2021; Beekmann et al., 2015). In addition to its effects on sebum production, quercetin may inhibit the hyperproliferation of sebaceous gland cells and promote their differentiation through the modulation of cell cycle-related proteins and key signaling pathways, including Wnt/β-catenin (Sferrazza et al., 2020). This action supports the restoration of normal sebaceous gland function and reduces excessive sebum secretion.

#### 4.3.2 Regulation of androgen receptor signaling

Androgens promote sebaceous gland secretion by binding to androgen receptors on sebaceous gland cells. Quercetin can either compete with androgens for binding to the androgen receptor or modulate the transcriptional activity of the androgen receptor, reducing the stimulatory effect of androgens on the sebaceous glands, and thus decreasing the amount of sebum secreted. Quercetin was found to inhibit androgen-induced expression of genes related to lipid synthesis in sebaceous gland cells, and quercetin may also inhibit the conversion of testosterone to dihydrotestosterone (DHT) by suppressing 5α-reductase activity, consequently reducing DHT’s stimulatory effect on sebaceous gland cells, decreasing the secretion of sebum, and ameliorating the symptoms of acne (Ghafouri-Fard et al., 2021a).

However these studies of sebaceous gland cells may be limited by the complexity and heterogeneity of the cells, which makes it difficult to resolve the spatial and molecular relationships of sebaceous gland cells at different stages of differentiation. In addition, the lack of specificity and efficiency of Cre drivers complicates genetic fate mapping studies on sebaceous glands. The effects of quercetin on sebaceous gland cells have mainly been performed *in vitro* or in mouse models, lacking sufficient evidence for effects on mast cells in obese patients or patients with abnormal glucose metabolism. Nevertheless, considering the crucial role of sebaceous gland hypersecretion in the pathogenesis of acne, and given that multiple studies have confirmed the regulatory effects of quercetin on sebaceous gland function from various perspectives, we believe that this set of data still holds potential validity. Future studies are needed to investigate how quercetin regulates sebaceous gland function in different physiologic and pathologic states and how these regulations affect acne development. There is also a need to develop new models and methods to better understand the heterogeneity and differentiation processes of sebaceous gland cells and how they respond to different physiological and pathological signals.

### 4.4 Antimicrobial effects of quercetin

#### 4.4.1 Inhibition of *C. acnes*

*C. acnes* is a Gram-positive, facultative anaerobe that predominantly resides within the sebaceous glands of hair follicles, where it is the most abundant microorganism (Fitz-Gibbon et al., 2013). Extensive research has established *Staphylococcus epidermidis (S. epidermidis)*, *Staphylococcus aureus (S. aureus)*, and *C. acnes* as key contributors to acne pathogenesis. Consequently, the effective inhibition of these acne-causing bacteria has become a crucial therapeutic strategy (Dreno et al., 2024; Dréno et al., 2020). Quercetin has been shown to significantly suppress *C. acnes*-induced pro-inflammatory cytokine production in HaCaT, THP-1, and RAW 264.7 cell lines, while also reducing TLR-2 expression and the phosphorylation of p38, ERK, and JNK MAPK in *C. acnes*-stimulated HaCaT and THP-1 cells. Additionally, quercetin inhibits MMP-9 mRNA levels *in vitro* across these cell lines exposed to *C. acnes*. *In vivo*, intradermal injection of *C. acnes* into mouse ears induced skin erythema, swelling, and granulomatous reactions, all of which were significantly attenuated by quercetin treatment, resulting in reduced ear thickness and inflammation (Lim et al., 2021). Moreover, quercetin has been found to induce apoptosis in *C. acnes*, thereby limiting bacterial overgrowth. It achieves this by activating caspase-3 and caspase-9, as well as modulating apoptosis-related genes such as Bax and Bcl-2 (Ghafouri-Fard et al., 2021b). Beyond its direct antimicrobial effects, quercetin also modulates the metabolic profile of *C. acnes*, reducing the release of harmful substances such as bacterial endotoxins while promoting the production of beneficial metabolites like short-chain fatty acids. This dual action not only inhibits bacterial stimulation of sebaceous units but also improves the local microenvironment within the follicle, contributing to the overall therapeutic potential of quercetin in acne management. Despite the inhibitory effect of quercetin on *C. acnes*, the effects of its long-term use on bacterial resistance and skin microecology are still not fully explored, which is an important direction for future research.

#### 4.4.2 Synergistic antibacterial effects

Quercetin, a natural antimicrobial agent, exhibits a synergistic antimicrobial effect when combined with conventional antibiotics. For instance, when used in conjunction with tetracycline antibiotics, quercetin enhances the inhibition of *C. acnes*, possibly by altering bacterial cell membrane permeability, thereby facilitating the entry of the antibiotic into the bacterium, or by modulating bacterial mechanisms associated with drug resistance. This synergy can improve antibiotic efficacy, reduce required dosages and treatment duration, and mitigate the risk of developing antibiotic resistance. A study combining quercetin with isotretinoin reported that the optimized formulation, containing 52.11% ± 2.85% isotretinoin and 25.44% ± 3.18% quercetin, demonstrated increased skin penetration and enhanced steady-state flux, along with excellent hepatoprotective activity in animal models (Hosny et al., 2020). Additionally, quercetin, when combined with amoxicillin, has been found to reverse amoxicillin resistance in *S. epidermidis* (Siriwong et al., 2016). However, despite its synergistic effects with antibiotics *in vitro*, quercetin’s absorption and bioavailability in humans are relatively low, which may limit the practical application of its antimicrobial properties.

#### 4.4.3 Effects on skin and gut microbiota

The skin microbiota is a complex ecosystem comprising diverse microorganisms, including bacteria, fungi, and viruses, in conjunction with host skin tissues, cells, secretions, and microenvironments. The interplay between microbes, the host, and external microenvironments maintains the skin’s microecological balance (O'Neill and Gallo, 2018). Under normal conditions, species such as *S. epidermidis*, *C. acnes*, and *S. aureus* inhibit each other, contributing to this equilibrium. The gut microbiota also plays a crucial role in skin health by modulating immune responses and metabolic pathways. Dysbiosis of gut microbes can increase intestinal permeability, leading to systemic inflammation that adversely impacts the skin (Sánchez-Pellicer et al., 2022). Studies have demonstrated that individuals with acne exhibit significantly reduced gut microbial diversity (Mahmud et al., 2022). It was previously believed that the proliferation of *C. acnes* was one of the key factors in the pathogenesis of common acne. In contrast, recent studies have concluded that the microbiota associated with the pathophysiology of acne in acne patients include *C. acnes*, *S. epidermidis*, *S. aureus*, *Streptococcus pneumoniae*, *Enterobacteriaceae*, *and Klebsiella pneumoniae* (Fitz-Gibbon et al., 2013; Hsieh and Chen, 2011; Ritvo et al., 2011). It is the disruption of the skin’s microecological balance and conventional antibiotic therapy may lead to skin microbiota dysbiosis and antimicrobial resistance (Dreno et al., 2024; Dréno et al., 2020). Restoring the skin’s microecological balance and *C. acnes* diversity has emerged as a more effective strategy for treating acne. Quercetin, known for its broad-spectrum antimicrobial properties, has shown inhibitory effects against various bacteria, including methicillin-resistant S. aureus (Farhadi et al., 2019). Quercetin has been shown to exhibit broad-spectrum antimicrobial properties (Kumar et al., 2016). Its antibacterial mechanism mainly includes disrupting the structure of bacterial cell membranes, inhibiting bacterial DNA and protein synthesis, and interfering with bacterial energy metabolism in various aspects (Nguyen and Bhattacharya, 2022). The regulatory effects of quercetin on microorganisms are shown in Table 1. While quercetin inhibits *S. aureus*, its broad-spectrum antimicrobial activity primarily targets pathogenic strains associated with acne dysbiosis. By selectively reducing *C. acnes* and *S. aureus* overgrowth without significantly affecting commensal species like *S. epidermidis*, quercetin may restore microbial equilibrium. Furthermore, its anti-inflammatory properties mitigate the inflammatory cascade triggered by dysbiosis, indirectly supporting a healthier skin microbiome. Most current studies on the antimicrobial effects of quercetin are primarily based on *in vitro* experiments, typically conducted under specific bacterial strains and controlled conditions. We believe that this part of the data demonstrates the potential of quercetin in antimicrobial aspects. These results may not fully translate to human physiology or complex biological environments. Furthermore, existing research has not sufficiently explored the potential for microbiota disruption caused by quercetin, particularly with prolonged use, raising important concerns about its impact on the skin microbiota that warrant further investigation. Therefore, more *in vivo* studies and long-term observations are needed to determine its true antimicrobial effectiveness and safety, in order to exclude the potential non-specific effects that may exist in *in vitro* experiments.

**TABLE 1:** Mechanism of action of quercetin on microorganisms.

Microorganism name	*In vitro or in vivo*	Metabolite type	Concentration range	MIC or MBIC	Mechanism of action	Reference
*Staphylococcus aureus (S. aureus)*	*In vitro*	Quercetin	0–128 μg/mL	MBIC: 64 μg/mL	Quercetin could suppress biofilm formation without impairing the growth of *S. aureus*	Kang et al. (2022)
*Staphylococcus epidermidis (S. epidermidis)*	*In vitro*	Quercetin	0–1,000 μg/mL	MIC: 250 μg/mL	Inhibits Biofilm Formation by Decreasing the Production of EPS and Altering the Composition of EPS in *Staphylococcus* epidermidis	Mu et al. (2021)
*Cutibacterium acnes (C. acnes)*	*In vitro and in vivo*	Quercetin	*Vitro*: 0.01–1 μM	-	Quercetin inhibits skin inflammatory response induced by C. acnes by suppressing the production of inflammatory factors, inhibiting the activation of TLR-2 and MAPK signaling pathways, and suppressing MMP-9 expression	Lim et al. (2021)
*Methicillin-resistant Staphylococcus aureus (MRSA)*	*In vitro*	Quercetin	4–512 µg/mL	MBIC: 4 μg/mL	Inhibition of biofilm formation and expression of bacterial attachment genes	Liu et al. (2024)
*Escherichia coli (E. coli)*	*In vitro and in vivo*	Quercetin	*Vivo*: 0, 0.2, 0.4, and 0.6 g/kg of diet	MIC: 0.0082 μmol/mL	Destroying the structure and integrity of cell walls and cell membranes, leading to cell lysis, cell wall deformation, leakage of intracellular substances, etc.	Wang et al. (2018b)
*Pseudomonas aeruginosa (P. aeruginosa)*	*In vitro*	Quercetin	16 µg/mL	-	Inhibits biofilm formation and reduces the production of multiple virulence factors by inhibiting bacterial population sensing and reducing swimming motility	Ouyang et al. (2020)
*Listeria monocytogenes (L. monocytogenes)*	*In vitro*	Quercetin	0.1–0.8 mM	MBIC: 0.8 mM, MIC: 4.9 mM	Blocking biofilm formation, inhibiting its stress genes and virulence, and altering its physicochemical cellular properties	Vazquez-Armenta et al. (2020)
*Klebsiella pneumoniae (K. pneumoniae)*	*In vitro and in vivo*	Quercetin	*Vivo*: 50 mg/kg *Vitro*: 0.016–32 μg/mL	-	Disruption of cell walls and cell membranes	Lin et al. (2021)
*Streptococcus pneumoniae (S. pneumoniae)*	*In vitro*	Quercetin	0–200 μM	MBIC: 12.5 μM	Inhibits sortase A activity and disrupts biofilm formation	Wang et al. (2018a)
*Salmonella typhimurium (S. typhimurium)*	*In vitro*	Quercetin	50–500 μg/mL	MIC: 200 μg/mL	Disruption of the structure and permeability of the cell membrane, leading to leakage of electrolytes from the cell	Toushik et al. (2023)
*Candida albicans (C.albicans)*	*In vitro and in vivo*	Quercetin	-	MIC: 128 μM	Inhibits biofilm formation and induces apoptosis in yeast cells through mitochondrial dysfunction	Tan et al. (2023)
*Streptococcus mutans (S.mutans)*	*In vitro*	Nano-quercetin	32–128 μg/mL	MBIC: 128 μg/mL	Inhibits biofilm formation and interferes with bacterial community sensing systems	Pourhajibagher et al. (2022)
*Bacillus subtilis (B. subtilis)*	*In vitro and in vivo*	Quercetin loading CdSe/ZnS nanoparticles	*Vitro*: 1–20 μg/mL *Vivo*: 2 mg/kg	MIC: 6.5 μg/mL	Disrupts cell membrane structure, inhibits biofilm formation, and enhances antimicrobial activity against drug-resistant strains by loading on nanoparticles	Yang et al. (2017)
*Trichophyton rubrum (T.Rubrum)*	*In vitro*	Quercetin	-	MIC: 63 μg/mL	Downregulation of fatty acid synthase gene expression and reduction of ergosterol content leading to plasma membrane disruption	Bitencourt et al. (2013)
*Aspergillus flavus (A.flavus)*	*In vitro*	Quercetin	50–800 μg/mL	MIC: 505 μg/mL	Quercetin inhibits the proliferation and aflatoxin biosynthesis by regulating the expression of development-related genes and aflatoxin production-related genes	Li et al. (2019)
*Herpes Simplex Virus (HSV-1)*	*In vitro*	Quercetin	0–30 l g/ml	-	Inhibition of viral polymerase activity reduces viral infectivity and intracellular replication	Lee et al. (2017)
*Influenza A Virus (H1N1)*	*In vitro*	Quercetin-derived carbonized nanogels	1–10 l g/ml	MIC: 4 l g/ml	Inhibits neuraminidase activity and prevents viruses from entering cells	Lin et al. (2022)
Hepatitis B Virus *(HBV)*	*In vitro*	Quercetin	50–200 μM	-	Reduces HBsAg and HBeAg secretion, inhibits HBV genome replication	Hao et al. (2024)
*Hepatitis C Virus (HCV)*	*In vitro*	Quercetin	25–125 μM	25 μM	Inhibits NS3 protease activity, reduces NS5A and HSP70 levels, blocks IRES-mediated translation	Khachatoorian et al. (2012)
*Human Cytomegalovirus (HCMV)*	*In vitro*	Quercetin	<25 μM	MIC: 4.8 ± 1.2 μM	Inhibits the production of Immediate Early Protein and Early Protein	Cotin et al. (2012)
*Severe Acute Respiratory Syndrome Coronavirus (SARS-CoV)*	*In vitro*	Quercetin	-	-	Inhibits 3CLpro and PLpro proteins, interferes with S protein binding to ACE2 receptor	Derosa et al. (2021)
Human Immunodeficiency Virus *(HIV-1)*	*In vitro*	Quercetin	-	-	Inhibits HIV-1 integrase and topoisomerase II activity, reactivates latent HIV-1 gene expression	Fesen et al. (1993)
Enterovirus 71 *(EV71)*	*In vitro and in vivo*	Quercetin	3–100 μM	-	Induces apoptosis, inhibits viral RNA and protein synthesis, inhibits 3C protease activity	Yao et al. (2018)
Respiratory Syncytial Virus *(RSV)*	*In vitro*	Quercetin	0–25.6 μM	-	Binds to RSV’s Non-Structural Protein 1 (NS1) and M2-1 protein, inhibiting viral replication	Gomes et al. (2016)
*Ebola Virus (EBOV)*	*In vitro*	Quercetin	3–100 μM	MIC: 7.4 μM	Inhibits VP24 protein’s anti-interferon function, blocking viral replication	Fanunza et al. (2020)

### 4.5 Skin barrier repair effects of quercetin

The pathogenesis and progression of acne are often associated with impaired skin barrier function. Misuse of topical anti-acne medications, excessive cleansing, and certain cosmetics can exacerbate this impairment, leading to increased transepidermal water loss and heightened vulnerability to external stimuli and infections (Takei et al., 2013). This damage to the skin barrier at lesion sites intensifies the inflammatory response and worsens the severity of acne lesions. Therefore, restoring and maintaining skin barrier function is a critical component of effective acne treatment. Quercetin has been shown to enhance skin barrier function by promoting keratinocyte proliferation and differentiation, and by stabilizing intercellular junctions, thereby preventing the invasion of external pathogens (Fitz-Gibbon et al., 2013; Kim et al., 2014). It has been demonstrated that quercetin improves skin barrier function through the activation of peroxisome proliferator-activated receptor (PPAR)-α and the inhibition of inflammatory cytokines. Moreover, quercetin 3-O-β-D-glucuronide has been found to enhance skin hydration by upregulating the expression of genes related to moisturization in keratinocytes, such as transglutaminase-1 (TGM-1) and hyaluronan synthase (HAS)-1 (Ha et al., 2021). Through these mechanisms, quercetin increases moisture content and lipid layer thickness on the skin surface by upregulating the expression of natural moisturizing factors (NMF) and lipid synthesis-related genes. This results in improved skin hydration and defense capabilities, thereby strengthening the skin barrier, reducing the penetration of external irritants, and protecting against inflammation and infection. Collectively, these effects facilitate the repair of acne lesions and contribute to the restoration of overall skin health. Notably, the restoration of skin barrier function may be influenced by a multitude of factors, including genetics, environment, and lifestyle, which are not fully accounted for in current studies. Therefore, additional human-based clinical data are needed to validate the efficacy of quercetin in restoring skin barrier function.

### 4.6 Anti-melanin and scarring effects of quercetin

The inflammatory process in acne often triggers excessive melanin production, leading to abnormal melanin deposition and resulting in post-inflammatory hyperpigmentation (PIH) in affected individuals (Davis and Callender, 2010). PIH is characterized by increased melanin production and distribution in both the epidermis and dermis (Del Bino et al., 2018). Melanin synthesis is closely linked to the expression and catalytic activity of key regulatory factors such as tyrosinase (TYR), tyrosinase-related protein 1 (TYRP1), and microphthalmia-associated transcription factor (MITF). TYR serves as the rate-limiting enzyme in melanin biosynthesis, while TYRP1 and MITF are critical for the regulation of melanin synthesis pathways. Quercetin’s effect on melanin synthesis regulators exhibits a degree of biphasic effects. Structurally, quercetin contains a catechol moiety capable of chelating Cu2+ at the active site of TYR, thereby inhibiting TYR activity and reducing melanin synthesis (Xue et al., 2011). A study demonstrated that quercetin, at concentrations of 1–10 μmol/L, inhibited the expression of TYR, TYRP1, and MITF in mouse B16 melanoma cells in a dose-dependent manner (Karadeniz et al., 2023). Conversely, another investigation reported that higher concentrations (1–50 μmol/L) of quercetin upregulated these same regulators, leading to increased melanin content (Liu et al., 2023). The paradoxical effects of quercetin on melanin synthesis may be dose-dependent, potentially mediated through bidirectional modulation of the P38MAPK-ERK signaling pathway, which in turn influences downstream expression of TYR, TYRP1, and MITF. Some studies suggest that quercetin can inhibit melanogenesis by blocking tyrosinase activity, a critical enzyme in the pigmentation process (Fan et al., 2017), while others report that quercetin can enhance tyrosinase activity and promote melanogenesis at different concentrations (Niu and Aisa, 2017). Experimental data suggest that quercetin concentrations exceeding 20 µM generally decrease melanin production, while concentrations between 10 and 20 µM may increase melanin content. However, higher concentrations (50–100 µM) also decrease melanin levels, though this effect may be associated with increased cytotoxicity, which can be mitigated by combining quercetin with vitamin C and arbutin (Choi and Shin, 2016). The relationship between the antimelanogenic effect of quercetin and its cytotoxicity needs to be further investigated, particularly in the context of long-term use or high concentrations. Balancing therapeutic efficacy with safety remains a critical challenge to address. The antimelanogenic effects of quercetin are also influenced by the position of hydroxyl groups and the type and position of sugar residues in quercetin derivatives (Yamauchi et al., 2013; Yamauchi et al., 2014). Additionally, quercetin’s anti-inflammatory properties contribute to reducing inflammation-induced melanogenesis. These multifaceted mechanisms suggest that quercetin not only holds promise for the treatment of acne but also for managing acne-associated hyperpigmentation. However, existing research faces several challenges. The dual effects of quercetin on melanogenesis, its dose-dependent responses, cytotoxicity, and the variability of its effects across individuals require further systematic investigation to resolve.

In the context of acne-induced pathological scarring, advances in understanding immunopathology have revealed that inflammatory cells play a pivotal role by directly secreting growth factors that activate fibroblasts and by releasing pro-inflammatory mediators that drive pathological scar formation. T helper cells contribute to scar formation through the secretion of IL-4 and IL-13, while regulatory T cells exert an anti-inflammatory effect by producing IL-10 and prostaglandin E2, which inhibit fibrosis (Xu et al., 2024). Quercetin, with its potent anti-inflammatory properties, has shown promise in reducing inflammation in scarred areas. Studies have demonstrated its potential in scar treatment, with evidence indicating that quercetin significantly inhibits hyperproliferation of keloidal fibroblasts and collagen deposition, thereby controlling and minimizing keloid formation (Moravvej et al., 2019). These findings are further supported by multiple investigations confirming quercetin’s inhibitory effects on keloid fibroblast proliferation and cell cycle progression (Zhou et al., 2017; Fu et al., 2020). For instance, quercetin has been found to suppress the insulin-like growth factor (IGF) signaling pathway and collagen expression, reducing keloid fibroblast proliferation in a dose-dependent manner (Phan et al., 2003). Further studies have demonstrated that quercetin inhibits proliferative scar formation primarily by targeting the Collagen Type III (COL-III), α-smooth muscle actin (α-SMA), and TGF-β1/Smad pathways (Ni et al., 2022). These mechanisms suggest that quercetin exerts multiple effects in antifibrosis; however, there is some inconsistency in the signaling pathways it modulates across studies. For instance, the effects of quercetin on the TGF-β1 and Smad pathways vary, indicating that its action on these pathways may be influenced by experimental conditions or model systems. These findings highlight the need for systematic research to evaluate how quercetin modulates these signaling pathways and how its effects may differ across various physiological and pathological contexts. Additional investigations have explored synergistic approaches, such as combining quercetin with vitamin D3, which reduces keloid fibroblast proliferation and collagen synthesis while inducing apoptosis (Mathangi Ramakrishnan et al., 2015). Moreover, studies suggest that quercetin can overcome keloid resistance to radiotherapy by inhibiting hypoxia inducible factor-1 (HIF-1) expression and modulating the PI3K/AKT pathway, reducing AKT phosphorylation, and thereby enhancing the therapeutic efficacy of radiotherapy in keloid scars (Si et al., 2018). However, studies on the combination of quercetin with other therapeutic modalities, such as laser treatment or topical medications, remain limited, and the understanding of its interactions and potential synergistic effects with these therapies is still insufficient. Further research is required to validate the efficacy and safety of quercetin in combination with other treatments in clinical settings. In summary, quercetin shows considerable potential in the treatment of acne scars through its multifaceted effects, including anti-inflammatory, antifibrotic, and cell signaling modulation, ultimately contributing to the improvement of skin appearance and function.

## 5 Potential applications of quercetin in acne vulgaris treatment

**FIGURE 1:**
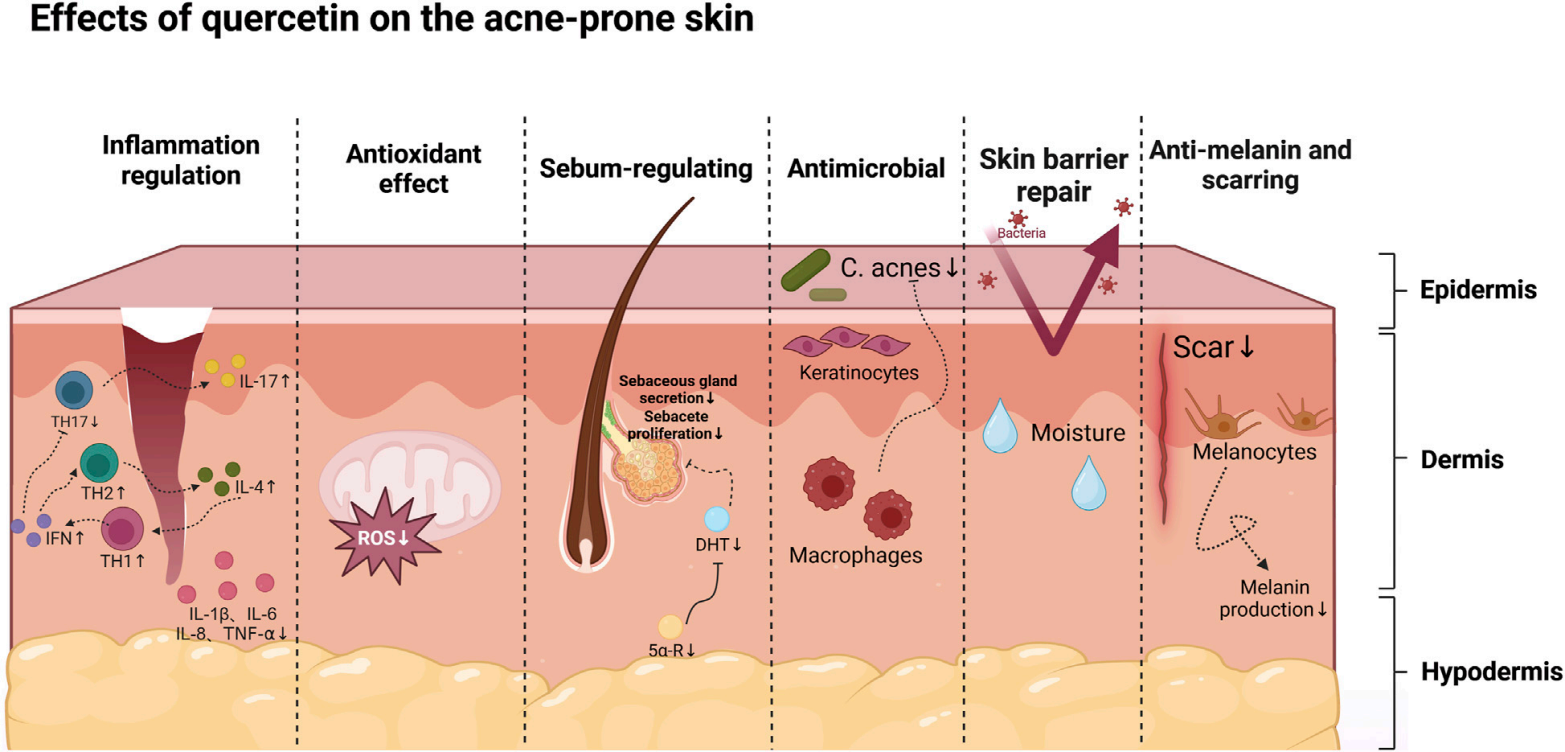
Summary of the effect of quercetin on acne-prone skin, created using Biorender.com.

**FIGURE 2:**
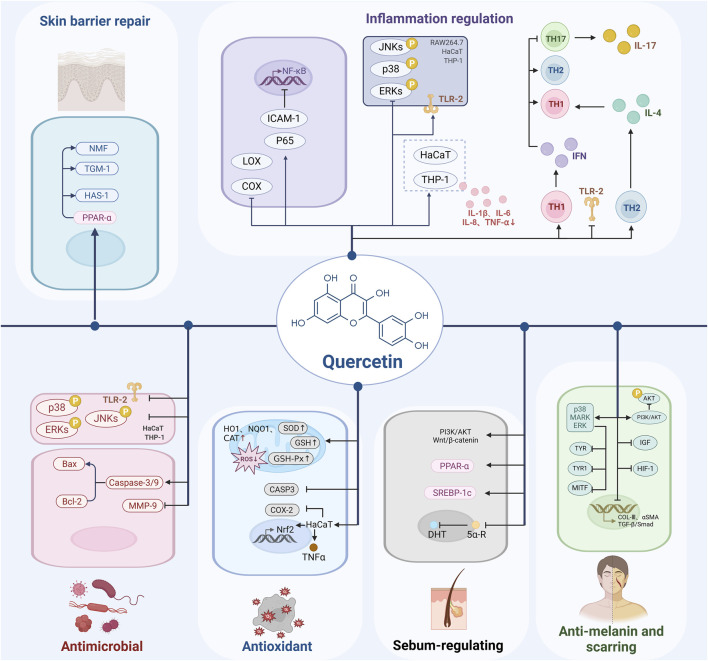
Schematic diagram of the mechanism of action of quercetin in the treatment of acne, created using Biorender.com.

As a natural plant metabolite, quercetin is widely available as a glycoside in fruits, vegetables, and other plants, offers a safer alternative to synthetic drugs, with fewer side effects. Its natural origin makes quercetin particularly appealing as a therapeutic option, especially for patients who are resistant to or experience adverse reactions to conventional treatments. Beyond its well-documented inflammation regulation, antioxidant, and antibacterial properties, quercetin regulates sebum secretion, repairs the skin barrier, resists melanin and scarring, and interacts with a diverse array of molecular targets (Figures 1, 2). This multifaceted mechanism of action enables quercetin to address the complex pathogenesis of acne, presenting a promising comprehensive treatment approach.

It is important to note that although we have found the multi-target effects of quercetin in the treatment of acne vulgaris, the interrelationships and synergistic mechanisms among these targets remain incompletely elucidated. Future research should delve deeper into the multi-targeted mechanisms of quercetin and its network regulatory relationships to provide a theoretical basis for developing more precise and effective therapeutic strategies. As shown in Table 2, quercetin’s effects demonstrate dose-dependent characteristics within specific concentration ranges, indicating high pharmacological relevance; however, current research is predominantly confined to *in vitro* cell models, failing to fully capture its physiological complexity in humans. The polyphenolic structure of quercetin predisposes it to non-specific interactions in simplified experimental systems (e.g., cell-free systems or high-concentration models), such as redox cycling, protein binding, and metal ion chelation, which may overestimate its apparent activity. To mitigate such artifacts, existing studies have employed dose-response experiments, comparative analyses with known pharmacological agents, and rigorously designed control experiments (e.g., exclusion of polyphenol-related artifacts) to delineate concentration ranges where quercetin’s effects are most likely target-specific. Collectively, while non-specific effects cannot be entirely ruled out, current data strongly suggest that observed effects predominantly stem from its pharmacological activity rather than non-specific interactions. Future research should adopt the following strategies for validation: 1) conduct additional *in vivo* and clinical studies under physiologically relevant conditions to clarify its true pharmacological actions and minimize limitations of *in vitro* models; 2) leverage advanced technologies such as single-cell sequencing and gene editing to precisely dissect quercetin’s cellular and molecular targets and signaling pathways, thereby distinguishing specific from non-specific effects. Moreover, discrepancies between the pathogenesis of acne in animal models and humans create uncertainties in translating these findings into clinical practice. As of the current registry on the clinical trials website (clinicaltrials. gov), approximately 111 clinical studies investigating quercetin have been registered, spanning diverse therapeutic areas including cardiovascular diseases, cancer, COVID-19, chronic obstructive pulmonary disease, and hyperuricemia. Among these, nine trials specifically focus on evaluating the anti-inflammatory effects of quercetin. Although there are no direct clinical trial data to support the efficacy and safety of quercetin in the treatment of acne, it can be hypothesized that it has some clinical feasibility in the treatment of acne based on its known bioactivity and potential for use in other inflammatory diseases. Future research should prioritize large-scale, multicenter clinical trials to evaluate the efficacy and safety of quercetin, both as a monotherapy and in combination regimens, for treating acne vulgaris. Such trials should encompass diverse patient populations, including variations in age, gender, and acne severity, to thoroughly assess therapeutic outcomes and potential adverse effects. Determining the optimal dosage, treatment duration, and target patient groups will be critical for clinical implementation. Additionally, investigations should focus on recurrence rates following quercetin treatment and its long-term impact on patients’ quality of life, ensuring sustained therapeutic benefits and safety.

**TABLE 2:** Overview of the biological effects of quercetin and its derivatives in different cell lines.

Type of cell	Metabolite type	Group	Dosage	Treat time	Findings	References
RAW264.7 cells	Quercetin (4H-1-benzopyran-4-one, 2-(3,4-dihydroxyphenyl)-3,5,7-trihydroxy-flavon), quercitrin (2-(3,4-dihydroxyphenyl)-5,7-dihydroxy-4-oxo-4H-chromen-3-yl6-deoxy-alpha-L-mannopyranoside)	LPS + PBS (model group, LPS: 2 μg/mL), LPS + DEX (positive control group, LPS: 2 μg/mL, DEX: 10 μg/mL), LPS + LQCT (LPS: 2 μg/mL, quercetin: 0.03 μg/mL), LPS + MQCT (LPS: 2 μg/mL, quercetin: 0.3 μg/mL), LPS + HQCT (LPS: 2 μg/mL, quercetin: 3 μg/mL), LPS + LQTR (LPS: 2 μg/mL, quercitrin: 0.045 μg/mL), LPS + MQTR (LPS: 2 μg/mL, quercitrin: 0.45 μg/mL), LPS + HQTR (LPS: 2 μg/mL, quercitrin: 4.5 μg/mL)	Quercetin: 0.03–15 μg/mL, quercitrin: 0.045–22.4 μg/mL	24 h	The concentration of NO can be significantly inhibited by quercetin and quercitrin. In LPS stimulated RAW264.7 cells, TNF - α, IL-1 β, and IL-6 decreased after intervention with quercetin and quercitrin, and the trend of ROS changes was similar to that of inflammatory factors	Tang et al. (2019)
HaCaT, HDMECs	Quercetin	Control group, TNF-α group, Quercetin+TNF-α group	100 μM	24 h	Quercetin significantly inhibited TNFα- and LL37-induced TNF-α, IL-6, IL-1β, CXCL1, CXCL2, and CCL3 expression in HaCaT cells	Meng et al. (2024)
HaCaT, THP-1 and RAW 264.7 cells	Quercetin	Control group, *C. acnes* group, Quercetin treated groups, DEXA group	0.01, 0.1 and 1 μM	Quercetin pretreatment time was 4 h, followed by *C.acnes* co stimulation for 18 h	Quercetin demonstrated no cytotoxicity on HaCaT, THP-1, and RAW 264.7 cells, while significantly inhibiting the production of pro-inflammatory cytokines, including IL-6, IL-8, IL-1β, and TNF-α, in these cells upon stimulation with *C. acnes*. Additionally, quercetin reduced the mRNA expression levels of TLR-2 and MMP-9 in *C. acnes*-stimulated HaCaT and THP-1 cells	Lim et al. (2021)
HaCaT and B16F10 cells	Quercetin 3-O-β-D-glucuronide (Q-3-G)	Control group, Q-3-G treated group, UVB irradiation group, H_2_O_2_ treatment group	0–20 µM	HaCaT: 24 h, B16F10 cells: 48 h	Q-3-G effectively protects HaCaT cells from UVB-induced cell death and concentration-dependently inhibits COX-2 and TNF-α mRNA expression. It enhances skin hydration by increasing FLG and TGM-1 expression. Exhibiting strong antioxidant activity, Q-3-G also upregulates Nrf2 expression. In B16F10 cells, it inhibits α-MSH-induced melanin secretion. Its skin protective effects are mediated through the activation of AP-1 and NF-κB signaling pathways	Ha et al. (2021)
B16F10 cells	Quercetin 3-O-Galactoside (Q3G)	α-MSH-stimulated B16F10 cells, unstimulated B16F10 cells, α-MSH-stimulated B16F10 cells treated with Q3G	1 μM–10 μM	24 h	Q3G decreased the α-MSH-stimulated increase in the mRNA levels of MITF, TYR, TRP-1, and TRP-2 in a dose-dependent manner	Karadeniz et al. (2023)
B16 melanoma cells, PIG3V melanocytes	Quercetin 3-O-(6″-O-E-caffeoyl)-β-D-glucopyranoside (coded as CC7)	Negative control group, CC7 treatment group, CC7 combined with specific signaling pathway inhibitors treatment group	1–50 μM	48 h	CC7 significantly increased the expression of TYR, TRP-1, TRP-2, and MITF. These results suggest that CC7 promotes the melanin content and tyrosinase activity through the upregulation of MITF and its downstream three melanogenesis-related enzymes in B16 cells	Liu et al. (2023)
Human hypertrophic scar fibroblasts (HSFb)	Quercetin	Control group, model group, and quercetin treatment group	125, 250, 500 μmol/L	48 h	Que mainly inhibits the proliferation of HSFb by suppressing the expression of COL-III, α - SMA, TGF - β 1, Smad2, and promoting the expression of Smad7, thereby suppressing the excessive deposition of extracellular matrix and activating the TGF - β 1/Smad pathway, and exhibiting a concentration dependent effect	Ni et al. (2022)
Keloid fibroblasts and keratinocytes	Quercetin	Vitamin D3 treatment group, quercetin treatment group, and control group	5–50 μg/ml	48 h	After quercetin treatment, collagen I expression was reduced, while caspase-3 expression was increased, suggesting a potential enhancement of apoptosis and a decrease in collagen synthesis	Mathangi Ramakrishnan et al. (2015)
Keloid fibroblasts and normal fibroblasts	Quercetin	Normal fibroblast group, keloid fibroblast group, ionizing radiation treatment group, and quercetin treatment group	20, 40, 80 μmol/L	24 h	Quercetin exerted an inhibitory effect on both Akt phosphorylation and HIF-1α accumulation, which suggests that the PI3K/Akt pathway might be implicated in the suppression of HIF-1α by quercetin	Si et al. (2018)

The medical application of quercetin is constrained by its poor water solubility, high metabolic rate, low oral bioavailability, and rapid clearance from the body (Sah et al., 2023; Panthi et al., 2023). A deeper understanding of quercetin’s pharmacodynamics, pharmacokinetics, and strategies to enhance its bioavailability is essential. Various nanotechnology-based drug delivery systems have been developed to improve its stability, absorption efficiency and therapeutic efficacy. Polymer-based nanoparticles, such as those synthesized from poly(lactic-co-glycolic acid) (PLGA), polylactic acid (PLA), polysaccharides (alginate and chitosan), inorganic materials (e.g., silica), or proteins, improve quercetin’s solubility and stability while enabling sustained or targeted release, minimizing gastrointestinal degradation, and enhancing systemic absorption (A et al., 2018, Kumar et al., 2015). Lipid-based systems, including liposomes composed of cholesterol and phospholipids, protect quercetin from rapid metabolism by encapsulating it within lipid bilayers. These structures facilitate direct cellular uptake via membrane fusion, modify biodistribution, and prolong release kinetics, thereby boosting bioavailability (Toniazzo et al., 2017; Seong et al., 2018). Nanoemulsions, stabilized by surfactants in colloidal biphasic systems (oil-in-water or water-in-oil), leverage natural milk protein surfactants to achieve enhanced solubility, bioaccessibility, and stability (Chen et al., 2018). Solid dispersions, which embed hydrophobic quercetin within hydrophilic carriers, improve its dispersibility and gastrointestinal dissolution (Gilley et al., 2017). Nanostructured lipid carriers (NLCs), combining solid and liquid lipids in surfactant-stabilized aqueous solutions, offer high stability, low cytotoxicity, and biocompatibility. NLCs protect quercetin from oxidation, enable organ-specific targeting (e.g., liver or lung accumulation), and prolong drug release (Khursheed et al., 2020). In acne therapy, nanoengineered quercetin formulations demonstrate superior efficacy compared to free quercetin. For instance, quercetin-loaded vitamin C nanoparticles exhibit enhanced antibacterial activity against *C*. *acnes* (Amer et al., 2020). Alginate/chitosan nanoparticles (ALG/CSNPs) and quercetin-loaded variants (Q-ALG/CSNPs) have been developed, which outperformed free quercetin in antimicrobial and antioxidant assays *in vitro*, highlighting their potential as advanced delivery systems (Nalini et al., 2022). Chitosan nanofiber patches (CSQCs), fabricated via solution blow spinning (SBS), integrate quercetin into polylactic acid (PLA) bilayer nanofiber functional patches (CSQC/PLA) to address antibiotic resistance and skin irritation in acne treatment. These patches demonstrated antibacterial, anti-inflammatory, and wound-healing properties (Yang et al., 2024). Similarly, electrospun polyvinyl alcohol/quercetin/essential oil composite nanofibers have been developed, showing potent anti-*C. acnes* activity and fibroblast biocompatibility, underscoring their promise as topical acne therapeutics (Amer et al., 2022). Collectively, nanoformulations overcome quercetin’s pharmacokinetic limitations, enabling targeted delivery and improved therapeutic outcomes. Future research should focus on elucidating structure-activity relationships, molecular mechanisms, and optimizing advanced systems such as micelles, microemulsions, microgels, and next-generation liposomes to further enhance stability, absorption, and clinical applicability (Manzoor et al., 2021; Tomou et al., 2023).

The UK-based Brand Essence Market Research reports that the quercetin market, in terms of revenue, is expected to reach USD 4. 6 billion by 2027, growing at a CAGR of 6. 53% from 2021 to 2027 (Brand Essence Market Research, 2022), with the major factor driving the growth of the quercetin market being its increasing use in dietary supplements. Currently, several quercetin-related pharmaceuticals or nutraceuticals have been marketed domestically and internationally, covering the forms of capsules, complexes, and herbal preparations. Examples include: Pure Encapsulations Quercetin (containing 500 mg of quercetin per serving), promoted to support cardiac metabolism, cellular, and immune health (Pure Encapsulations, n.d.); Elexir pharma C-vitamin komplex which contains 500 mg of vitamin C, 125 mg of quercetin, rutin, bioflavonoids produced in honeysuckle, and 75 mg of rosehip powder per tablet, a combination of nutrients that the brand claims helps the body neutralize free radicals, promotes the proper functioning of the immune system, supports the proper production of collagen, and reduces the sensation of fatigue and lethargy (Elexir Pharma, n.d.). Although these quercetin-related products do not directly target acne, their anti-inflammatory, antioxidant, and antimicrobial properties make them potentially marketable in acne treatment. The share of quercetin-containing formulations in the acne treatment market is expected to expand further as consumer demand for natural ingredients and safe medications increases.

The multi-targeted mechanisms of quercetin in treating acne vulgaris highlight its potential for development as a single-drug formulation. Quercetin can be formulated into various dosage forms, such as oral tablets or capsules, as well as topical gels and creams. Oral formulations may exert systemic effects by modulating sebaceous gland function, providing anti-inflammatory benefits, and inhibiting *C. acnes*. Topical formulations can directly target affected skin areas, addressing follicular keratinization abnormalities and localized inflammation. In the drug development process, optimizing the formulation of quercetin to enhance its bioavailability and stability is crucial, accompanied by rigorous clinical trials to validate its efficacy and safety. Given the complex pathogenesis of acne vulgaris, combining quercetin with other therapeutic agents may yield superior outcomes. For example, Hosny et al. (2020) successfully developed a self-nanoemulsion drug delivery system combining isotretinoin and quercetin. *In vivo* studies in experimental animals demonstrated enhanced hepatoprotective activity compared to other formulations and commercial products, representing a significant breakthrough in the safe treatment of acne vulgaris. When combined with antibiotics, quercetin, as previously discussed, can reduce antibiotic dosages and the risk of resistance while improving therapeutic efficacy. Combination regimens should be individualized based on factors such as disease severity, patient age, and gender. Additionally, quercetin could be incorporated into functional skincare products for adjunctive therapy and prevention of acne vulgaris. For instance, quercetin-enriched cleansers, toners, and lotions could deliver antibacterial and anti-inflammatory effects during routine skincare. Cleansers containing quercetin could clean the skin while reducing bacterial load and inflammation. Toners and lotions could provide prolonged effects by regulating sebum secretion and mitigating skin inflammation. When incorporating quercetin into cosmetic formulations, its safety and stability must be carefully considered to ensure efficacy while avoiding adverse reactions such as skin irritation.

## 6 Conclusion

In summary, quercetin plays a pivotal role in acne treatment through its multifaceted mechanisms, including anti-inflammatory, antioxidant, antimicrobial, sebum-regulating, and immunomodulatory effects. Quercetin not only alleviates inflammation and oxidative stress in acne lesions but also regulates sebaceous gland secretion, inhibits the proliferation of *C*. *acnes*, and promotes the restoration of skin barrier function, thereby improving clinical symptoms and enhancing the quality of life for acne patients. Despite the progress made in understanding quercetin’s therapeutic potential for acne, several challenges remain. Its polyphenolic properties need to be strictly examined to avoid interference from non-specific effects. Future research should focus on elucidating the molecular mechanisms of quercetin and its interactions with other drugs. Additionally, larger and longer-term clinical trials are needed to validate the efficacy and safety of quercetin across different types and severities of acne. Key areas for future investigation include optimizing dosage, administration routes, and drug delivery systems, as well as exploring synergistic effects with other treatments. Furthermore, the safety and efficacy of quercetin in special populations, such as pregnant or lactating women and children, warrant careful consideration to ensure its safe clinical application. Quercetin holds promise as a novel therapeutic option for acne, offering a safer and potentially more effective treatment alternative for patients.

## Glossary

α-SMA: α-smooth muscle actin
AP-1: activator protein-1
Bax: BCL2-Associated X protein
Bcl-2: B-cell lymphoma-2
COL-III: Collagen Type III
DHT: dihydrotestosterone
ERK: extracellular regulated protein kinases
GSH: glutathione
GSH-Px: glutathione peroxidase
HaCaT: human immortalized keratinocytes
HAS-1: hyaluronan synthase-1
HDMECs: human dermal microvascular endothelial cells
HIF-1: hypoxia inducible factor-1
HO-1: heme oxygenase-1
ICAM-1: intercellular cell adhesion molecule-1
IGF: insulin-like growth factor
IL-1β: interleukin-1β
IL-4: interleukin-4
IL-6: interleukin-6
IL-17: interleukin-17
IFN-γ: Interferon-γ
JNK: c-Jun N-terminal kinase
MAPK: mitogen-activated protein kinases
MBIC: minimum biofilm inhibitory concentration
MIC: minimum inhibitory concentration
MMP-9: matrix metalloproteinase-9
mTOR: mammalian target of rapamycin
NF-κB: nuclear factor kappa-B
NMF: natural moisturizing factors
NQO1: NAD(P)H:quinone oxidoreductase 1
NRF2: nuclear respiratory factor 2
PI3K/AKT: phosphatidylinositol-3-kinase/protein Kinase B
PPAR-γ: peroxisome proliferator-activated receptor-γ
RAW 264.7: mouse leukemia cells of monocyte macrophage
ROS: reactive oxygen species
SOD: superoxide dismutase
SREBP-1c: sterol regulating element binding protein 1c
TNF-α: tumor necrosis factor-α
TGF-β: transforming growth factor-β
TGM-1: transglutaminase-1
THP-1: human myeloid leukemia mononuclear cells
TLR-2: Toll-like receptor-2
Wnt/β-catenin: Wingless/Int-1/β-catenin
